# A Multi-Model Combined Filter with Dual Uncertainties for Data Fusion of MEMS Gyro Array

**DOI:** 10.3390/s19010085

**Published:** 2018-12-27

**Authors:** Qiang Shen, Jieyu Liu, Xiaogang Zhou, Lixin Wang

**Affiliations:** Xi’an Research Institute of Hi-Tech, Hongqing Town, Xi’an 710025, China; shenq110@163.com (Q.S.); hebing110@163.com (X.Z.); johnson_outlook@163.com (L.W.)

**Keywords:** MEMS gyroscope, gyro array, set-membership filter, combined filter, multiple models, data fusion

## Abstract

The gyro array is a useful technique in improving the accuracy of a micro-electro-mechanical system (MEMS) gyroscope, but the traditional estimate algorithm that plays an important role in this technique has two problems restricting its performance: The limitation of the stochastic assumption and the influence of the dynamic condition. To resolve these problems, a multi-model combined filter with dual uncertainties is proposed to integrate the outputs from numerous gyroscopes. First, to avoid the limitations of the stochastic and set-membership approaches and to better utilize the potentials of both concepts, a dual-noise acceleration model was proposed to describe the angular rate. On this basis, a dual uncertainties model of gyro array was established. Then the multiple model theory was used to improve dynamic performance, and a multi-model combined filter with dual uncertainties was designed. This algorithm could simultaneously deal with stochastic uncertainties and set-membership uncertainties by calculating the Minkowski sum of multiple ellipsoidal sets. The experimental results proved the effectiveness of the proposed filter in improving gyroscope accuracy and adaptability to different kinds of uncertainties and different dynamic characteristics. Most of all, the method gave the boundary surrounding the true value, which is of great significance in attitude control and guidance applications.

## 1. Introduction

The advent of micro-electro-mechanical system (MEMS) gyroscopes have added motion sensing in consumer devices and special military applications [1,2,3]. This is because it has many excellent properties, including being lightweight and having a compact size, low power consumption, low cost, and ease of mass production. However, when compared to traditional gyroscopes, the MEMS gyroscopes are still not sufficiently accurate for many applications, such as space vehicles. 

To enlarge the application field of MEMS gyroscopes, it is necessary to improve their accuracy further while not restricting their advantages (e.g., some effective modeling and processing methods were proposed in References [4,5] to reduce temperature drift). Due to the large increase in cost and time consumption of the previous methods focusing on the device itself [6,7], the gyro array was presented with the development of multisensor information fusion technology. The gyro array technique was first proposed by Bayard and Ploen [8], who integrated four gyroscope outputs to obtain an optimal rate signal by data fusion. The gyro array technique made rapid progress after that [9,10,11].

The key to the gyro array technique is the estimate method. A Kalman filter and its variants and extensions are always used, which have been proven effective to some extent. The fusion method used in the works of Bayard and Pleon was the Kalman filter. Then, an extended Kalman filter to fuse multiple gyroscopes for accuracy improvement was presented by Stubberud [12]. After that, Chang, Xue, and Jiang at Northwestern Polytechnical University played an important role in promoting the development of gyro arrays. They constructed an array with six gyroscopes [13,14,15,16] and adopted an optimal Kalman filter to process their signal. Meanwhile, the performance of the gyro array was analyzed in detail. A 3 × 3 planar array configuration of MEMS gyroscopes was developed by Heera, and Kalman filtering with a constant gain matrix for a minimum rate estimate was implemented in Reference [17]. A new statistic, called the “Allan covariance” between two gyros, was introduced by Richard, and the gyro array model could be used to obtain the Kalman filter-based (KFB) virtual gyro [18].

The fusion methods mentioned above have been proven to improve the accuracy of data effectively. However, some difficulties still exist.

On the one hand, the Gaussian noise assumption in these classical techniques may render them invalid in some practical applications. When the Kalman filters are used for a gyro array, the uncertainty of noise statistical character may lead to degeneration in accuracy. An innovative alternative is to use the set-membership estimation approach, in which noises are assumed to be unknown but bounded [19,20,21]. For the set-membership approach, the estimate results are expressed as state feasible sets. Especially, the ellipsoidal set is an important way to describe the set-membership uncertainty [22,23]. Due to the complexity of the environment and the variation of the motion status, the stochastic uncertainty and the set-membership uncertainty always exist simultaneously in the outputs of the gyroscopes. However, the existence of Gaussian noise always makes the choice of the noise bounds too conservative and leads to accuracy degeneration in the set-membership estimations. In order to take advantage of each type of uncertainty, a combined filter with dual uncertainties is necessary.

On the other hand, the motion status of the carrier is always changing in applications, and the dynamic characteristic cannot be well represented by a single model. This may also impact the performance of a gyro array badly. To ensure reliable estimation results in the case of complex motion of the carrier, several models are used to describe the various motion statuses of the carrier. 

Therefore, the motivation of our research was to design an effective estimate fusion method that could solve the above two difficulties. To achieve this goal, a dual-noise acceleration model is presented to describe the rate signal instead of a random walk, and a multi-model combined filter with dual uncertainties is proposed to combine the MEMS gyro array readings. The proposed method in this paper has two advantages, which are the main contributions of this paper: (1) With two types of uncertainty, the proposed combined filter based on sets of densities can not only capture the true mean and error statistics, but can also obtain the guaranteed estimation bounds; and (2) the subfilters in the proposed algorithm can adaptively switch between a known set of models at each sampling instant to respond to the dynamic changes of the system in time.

In this paper, the gyroscope error model with dual uncertainties used in this research is established in Section 2. A brief introduction to the ellipsoidal sets and relevant properties is given in Section 3. Then, the multi-model combined algorithm is deduced in Section 4. Numerical experimental results are presented in Section 5. Conclusions are drawn in Section 6.

## 2. The Dual Uncertainties Model of a Gyro Array

The most common model for a target maneuver is the white-noise acceleration model [24]. It assumes that the target acceleration is an independent process, which is strictly white noise. It differs from the non-maneuver model only in the noise level: The white noise process w used to model the effect of the control input u has a much higher intensity than the one used in a non-maneuver model. Considering that the MEMS gyroscopes always have tiny sampling internally, this kind of model can be used to model attitude maneuver of the gyro or the gyro-installed carrier. The magnitude of the process noise is used to adjust the dynamic characteristics in the model. Due to the fact that both stochastic and set-membership uncertainties always exist simultaneously in the outputs of the gyroscopes, the angular acceleration is assumed to be an independent process with dual uncertainties. Thus, we call it a dual-noise acceleration model.

Assume that ω(t) is the angular velocity of the carrier in a certain coordinate direction: The kinematic equation of the dual-noise acceleration model is then given as

(1) $$\dot{\omega }(t)=w(t)+d(t),$$

where w(t) is a zero-mean Gaussian noise (stochastic uncertainty), and d(t) is the bounded noise (set-membership uncertainty).

**Figure 1:**
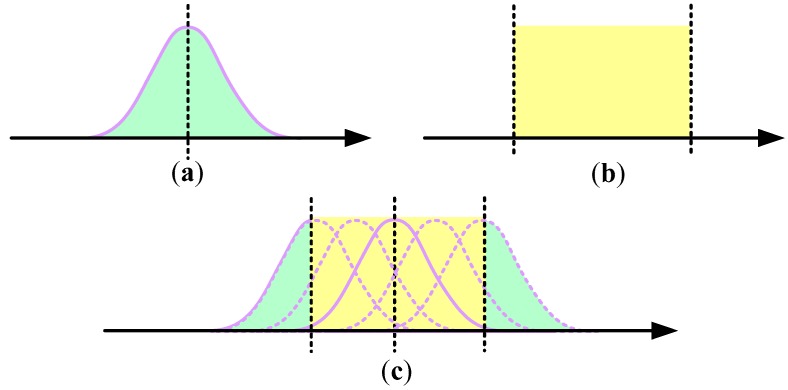
The model with dual uncertainties can be regarded as a set of Gaussian densities (e.g., the dual-noise acceleration model): (**a**) Stochastic model (w); (**b**) set-membership model (d); (**c**) dual uncertainties model ($\dot{\omega }$).

In fact, the dual uncertainties can be interpreted as a set bounded uncertainty incorporated into the prior stochastic uncertainty [25]. For the dual-noise acceleration model given in Equation (1), the angular velocity acceleration $\dot{\omega }$ is the sum of the zero-mean Gaussian noise $w∼N(0,\sigma^{2})$ with variance $\sigma^{2}$ and the bounded uncertainty d∈G⊂ℝ. Then $\dot{\omega }$ can be regarded as a random variable with mean d. The special thing is that it cannot be seen as a single distribution, but a set (i.e., $\left\{N(d,\sigma^{2})|d\in G\right\}$), as illustrated in Figure 1.

In the multidimensional case, it can be expressed as $\left\{N(d,C){|d\in G}^{n}\subset {\mathbb{R}}^{n},C\in {\mathbb{R}}^{n\times n}\right\}$. Then the state estimate is characterized by a set of probability densities through the combination of stochastic and set-membership uncertainties.

Our proposed method poses the gyroscope filtering problem as a hidden Markov model (HMM). Assume that the state vector is $x(t)={\left[\theta (t),\omega (t)\right]}^{T}$, where θ(t) is the attitude angle. Considering the dual uncertainties, the discrete-time state-space model for the gyroscope array in the carrier can then be described as

(2) $$x_{k}=F_{k}^{\left(i\right)}x_{k-1}+G_{k}^{\left(i\right)}(w_{k}^{\left(i\right)}+d_{k}^{\left(i\right)}),$$

(3) $$z_{k}=H_{k}^{\left(i\right)}x_{k}+v_{k}^{\left(i\right)}+e_{k}^{\left(i\right)},$$

with the mode transition governed by a Markov chain with probabilities

(4) $$P(m_{k}^{i}|m_{k-1}^{j})=p_{ji},$$

where $z_{k}$ is the measurement vector, and $F_{k}^{\left(i\right)}$, $G_{k}^{\left(i\right)}$, and $H_{k}^{\left(i\right)}$ are the state transition matrix, the noise matrix, and the observation matrix corresponding to the active model $m_{k}^{i}(i=1,2,\cdots ,r)$ at time k, respectively; $w_{k}^{\left(i\right)}∼N(0,Q_{k}^{\left(i\right)})$ is the stochastic part of the process noise; $v_{k}^{\left(i\right)}∼N(0,R_{k}^{\left(i\right)})$ is the stochastic part of the measurement noise, which are both assumed to be white Gaussian; and $d_{k}$ and $e_{k}$ are set-membership parts, which are assumed to be of unknown distribution, but bounded by

(5) $$D_{k}^{\left(i\right)}=\{d_{k}^{\left(i\right)}:{\left(d_{k}^{\left(i\right)}\right)}^{T}{\left(D_{k}^{\left(i\right)}\right)}^{-1}d_{k}^{\left(i\right)}\leq 1\},$$

(6) $${ℰ}_{k}^{\left(i\right)}=\{e_{k}^{\left(i\right)}:{\left(e_{k}^{\left(i\right)}\right)}^{T}{\left(E_{k}^{\left(i\right)}\right)}^{-1}e_{k}^{\left(i\right)}\leq 1\}.$$

In this research, six separate gyroscopes were taken to construct a gyroscope array. According to the model shown in Equation (1), the magnitude of the process noise directly affects the ability of the model to reproduce the dynamic characteristics of the input signal. In view of this situation, this paper designed a combined filter using multiple models with different process noise magnitudes and making the noise magnitude cover a certain range by interaction: Thereby the optimality of the estimation and the stability of the filter were guaranteed. Therefore, the model set with dual uncertainties designed in this paper is given by

(7) $$\begin{array}{l} F_{k}^{\left(i\right)}=\left[\begin{array}{cc} 1 & \Delta T\\ 0 & 1 \end{array}\right],G_{k}^{\left(i\right)}=\left[\begin{array}{c} 0\\ \Delta T \end{array}\right],H_{k+1}^{\left(i\right)}=\left[\begin{array}{cccccc} 0 & 0 & 0 & 0 & 0 & 0\\ 1 & 1 & 1 & 1 & 1 & 1 \end{array}\right],\\ Q_{k}^{\left(i\right)}=Q_{i},R_{k}^{\left(i\right)}=r^{2}CorrM,D_{k}^{\left(i\right)}=D_{i},E_{k}^{\left(i\right)}=e^{2}CorrM\\ i=1,2,3\cdots ,r \end{array}$$

where CorrM is a 6 × 6 correlation matrix of gyro array, $r^{2}$ is the Gaussian measurement noise variance of component gyroscopes, $e^{2}$ is a coefficient in the matrix defining the shape of the measurement noise set, and ΔT is the sampling interval. Obviously the differences between the models in Equation (7) are the values of $Q_{i}$ and $D_{i}$, which represent the level of the stochastic part and set-membership part of the process noise.

## 3. Ellipsoidal Set and Relevant Properties

When considering the set-membership uncertainty, the set operation is involved. In order to simplify the calculations, we confined the bounding feasible sets to the ellipsoids. Several definitions and lemmas about ellipsoid sets are given below, which were used in the derivation of the multi-model combined filter with dual uncertainties.

**Definition** **1.** *A bounded ellipsoid ℰ of ${\mathbb{R}}^{n}$ is defined by [23]*

(8) $$ℰ\left(a,M\right)=\{x\in {\mathbb{R}}^{n}:{\left(x-a\right)}^{T}M^{-1}(x-a)\leq 1\},$$

*where $a\in {\mathbb{R}}^{n}$ is the center of ℰ, and $M\in {\mathbb{R}}^{n\times n}$ is a positive-definite matrix that specifies its size and orientation.*

*Then $d_{k}$ and $e_{k}$ in Equations (5) and (6) are actually bounded by the ellipsoids $ℰ(0,D_{k}^{\left(i\right)})$ and $ℰ(0,E_{k}^{\left(i\right)})$, respectively.*

**Definition** **2.** *Given an ellipsoid $ℰ(a,M)(a_{1}\in {\mathbb{R}}^{n})$ and a full rank matrix $A\in {\mathbb{R}}^{n\times n}$, then {Ax:x∈ℰ(a,M)} can be expressed as Aℰ(a,M).*

**Lemma** **1.** *Given an ellipsoid $ℰ(a,M)(a_{1}\in {\mathbb{R}}^{n})$ and a matrix $A\in {\mathbb{R}}^{n\times n}$, then $Aℰ(a,M)=ℰ(Aa,AMA^{T})$.*

**Proof.**  According to **Definition 1**,

(9) Aℰ(a,M)={Ax:x∈ℰ(a,M)}.

Assume y=Ax and then $x=A^{-1}y$, and thus

(10) $${\left(A^{-1}y-a\right)}^{T}M^{-1}(A^{-1}y-a)\leq 1.$$

After some manipulations, Equation (10) becomes

(11) $${\left(y-Aa\right)}^{T}{\left(AMA^{T}\right)}^{-1}(y-Aa)\leq 1.$$

This means $y\in ℰ\;(A_{a},\;{\mathrm{AMA}}^{T})$. The proof is complete.  □

**Definition** **3.** *The Minkowski sum $\Psi_{s}$ of the two ellipsoids $ℰ\left(a_{1},M_{1}\right)$ and $ℰ\left(a_{2},M_{2}\right)$ is defined as*

(12) $$\Psi_{s}=\left\{x:x=x_{1}+x_{2},x_{1}\in ℰ\left(a_{1},M_{1}\right),x_{2}\in \left(a_{2},M_{2}\right)\right\},$$

*denoted as*

(13) $$\Psi_{s}=ℰ\left(a_{1},M_{1}\right)⊕ℰ\left(a_{2},M_{2}\right).$$

**Figure 2:**
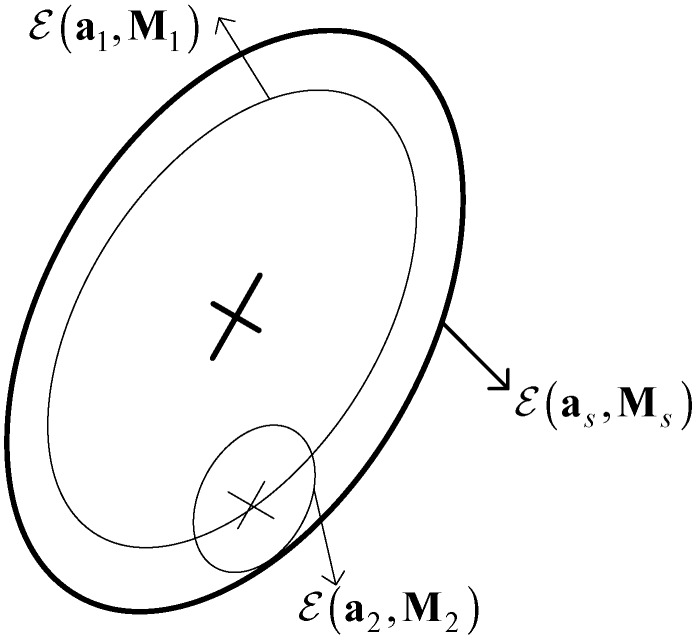
The Minkowski sum of two ellipsoids.

The ellipsoid satisfying $ℰ\left(a_{s},M_{s}\right)⊇\Psi_{s}$ is called the outer bounding ellipsoid of the Minkowski sum $\Psi_{s}$, as illustrated in Figure 2.

The following lemma is given to calculate the outer bounding ellipsoid of the Minkowski sum of two ellipsoids:

**Lemma** **2.** *∀p∈(0,+∞), $ℰ\left(a_{s},M_{s}\right)⊇ℰ\left(a_{1},M_{1}\right)⊕ℰ\left(a_{2},M_{2}\right)$, with $a_{s}=a_{1}+a_{2}$$\left(a_{1},a_{2}\in {\mathbb{R}}^{n}\right)$ and $M_{s}=\left(1+p^{-1}\right)M_{1}+\left(1+p\right)M_{2}$$\left(M_{1},M_{2}\in {\mathbb{R}}^{n\times n}\right)$.*

**Proof.**  See the works of Maksarov and Norton [22].

For a weighted Minkowski sum of several ellipsoids, the outer bounding ellipsoid is given by the following lemma.  □

**Lemma** **3.** *Given $\mu_{k}\in \mathbb{R}$ and the ellipsoids $ℰ\left(a_{k},M_{k}\right)$ ($k=1,2,\cdots r,r\in {\mathbb{R}}^{+}$), $\forall \alpha \in {\mathbb{R}}^{r}$, with $\alpha_{k}>0$ and $\sum_{k=1}^{r}\alpha_{k}=1$, the ellipsoid ℰ(a,M) with*

(14) $$a=\sum_{k=1}^{r}\mu_{k}a_{k},$$

(15) $$M=\sum_{k=1}^{r}\alpha_{k}^{-1}\mu_{k}^{2}M_{k},$$

*contains $\mu_{1}ℰ\left(a_{1},M_{1}\right)⊕\mu_{2}ℰ\left(a_{2},M_{2}\right)⊕\cdots ⊕\mu_{r}ℰ\left(a_{r},M_{r}\right)$.*

**Proof.**  According to **Lemma 1**, it can be easily obtained that

(16) $$\mu_{k}ℰ(a_{k},M_{k})=ℰ(\mu_{k}a_{k},\mu_{k}^{2}M_{k}).$$

Thus, the weighted Minkowski sum can be transformed into

(17) $$ℰ\left(\mu_{1}a_{1},\mu_{1}^{2}M_{1}\right)⊕ℰ\left(\mu_{2}a_{2},\mu_{2}^{2}M_{2}\right)⊕\cdots ⊕ℰ\left(\mu_{r}a_{r},\mu_{r}^{2}M_{r}\right).$$

On this basis and combined with relevant conclusions in Reference [23], a parametrized family of ellipsoids given by Equations (14) and (15) is obtained.  □

## 4. Multi-Model Combined Filtering with Dual Uncertainties (MMCF)

**Figure 3:**
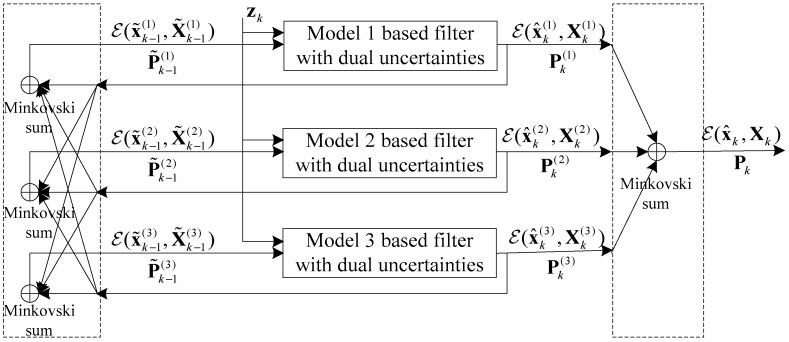
Structure of multi-model combined filtering with dual uncertainties (MMCF) algorithm (with three models).

Following the interacting multiple model (IMM) filter [26] merging strategy, the proposed multi-model state estimate combining dual uncertainties can be updated through 4 steps: First, the mixing probabilities and the reinitialized estimates for the corresponding mode-matched filter are calculated. Then the mixing estimates are used to input into the mode-matched filter new measurements and to update the estimates. Next, the updated mode probability is calculated. Finally, the overall estimates are found using moment matching. The main difference is that the set operation is involved in the proposed algorithm, and the output contains the covariance matrix $P_{k}$ and an ellipsoidal set $ℰ({\hat{x}}_{k},X_{k})$, which represents a set of means. The architecture of the MMCF algorithm is illustrated in Figure 3.

At time *k*, the MMCF algorithm can be given as follows.

### 4.1. Model-Conditioned Reinitialization

The predicted probability is given by

(18) $$\mu_{k,k-1}^{\left(i\right)}≜P\left\{m_{k}^{\left(i\right)}|Z^{k-1}\right\}=\sum_{j=1}^{r}p_{ji}\mu_{k-1}^{\left(j\right)},$$

where $\mu_{k-1}^{\left(j\right)}$ is the calculated probability for model $m_{k-1}^{j}$ at time k−1, and $Z^{k-1}=\left\{z_{1},z_{2},\cdots ,z_{k-1}\right\}$.

Then the mixing weight is calculated by

(19) $$\mu_{k-1}^{j|i}≜P\left\{m_{k-1}^{\left(j\right)}|m_{k}^{\left(i\right)},Z^{k-1}\right\}=p_{ji}\mu_{k-1}^{\left(j\right)}/\mu_{k,k-1}^{\left(i\right)}.$$

Let ${\hat{x}}_{k-1}^{\left(j\right)}$ be the state estimate of the subfilter for model $m_{k-1}^{j}$, and let $ℰ({\hat{x}}_{k-1}^{\left(j\right)},X_{k-1}^{\left(j\right)})$ and $P_{k-1}^{\left(j\right)}$ be the error characteristics of the state estimate for the set-membership uncertainty and the stochastic uncertainty, respectively.

Suppose that only the zero-mean Gaussian uncertainty exists (i.e., $X_{k-1}^{\left(j\right)}=0$): Then the mixing estimate and covariance matrix are

(20) $${\tilde{x}}_{k-1}^{\left(i\right)}=\sum_{j-1}^{r}\mu_{k-1}^{j|i}{\hat{x}}_{k-1}^{\left(j\right)},$$

(21) $${\tilde{P}}_{k-1}^{\left(i\right)}=\sum_{j=1}^{r}\left[P_{k-1}^{\left(j\right)}+({\tilde{x}}_{k-1}^{\left(j\right)}-{\hat{x}}_{k-1}^{\left(j\right)})({\tilde{x}}_{k-1}^{\left(j\right)}-{\hat{x}}_{k-1}^{\left(j\right)}{)}^{\prime }\right]\mu_{k-1}^{j|i}.$$

When considering the set-membership uncertainty, the mixing set of means therefore yields the Minkowski sum

(22) $${\tilde{X}}_{k-1}^{\left(i\right)}=\mu_{k-1}^{1|i}ℰ({\hat{x}}_{k-1}^{\left(1\right)},X_{k-1}^{\left(1\right)})⊕\mu_{k-1}^{2|i}ℰ({\hat{x}}_{k-1}^{\left(2\right)},X_{k-1}^{\left(2\right)})⊕\cdots ⊕\mu_{k-1}^{r|i}ℰ({\hat{x}}_{k-1}^{\left(r\right)},X_{k-1}^{\left(r\right)}).$$

Then the object in this step is to find an optimal ellipsoid $ℰ({\tilde{x}}_{k-1}^{\left(i\right)},{\tilde{X}}_{k-1}^{\left(i\right)})⊇{\tilde{X}}_{k-1}^{\left(i\right)}$. According to **Lemma 3**, there is a family of ellipsoids that meet this condition with a center ${\tilde{x}}_{k-1}^{\left(i\right)}$ and the matrix defining the shape

(23) $${\tilde{X}}_{k-1}^{\left(i\right)}=\sum_{j=1}^{r}{\left(\mu_{k-1}^{j|i}\right)}^{2}X_{k-1}^{\left(j\right)}/\alpha_{j}^{\left(i\right)},$$

where the parameter $\alpha_{j}^{\left(i\right)}>0$ and $\sum_{j=1}^{r}\alpha_{j}^{\left(i\right)}=1$. The parameters $\alpha_{j}^{\left(i\right)}$ are chosen to minimize the size of $ℰ({\tilde{x}}_{k-1}^{\left(i\right)},{\tilde{X}}_{k-1}^{\left(i\right)})$ by using the volume minimization criterion or trace minimization criterion. In this paper, the trace of ${\tilde{X}}_{k-1}^{\left(i\right)}$ was minimized due to the explicit expression of the parameter and avoidance of the nonlinear equation solution. Obviously, this criterion is favorable to computation efficiency. Then the parameter optimization problem is given by

(24) $$\begin{array}{l} Min:\mathrm{tr}({\tilde{X}}_{k-1}^{\left(i\right)})\\ S.T.\{\begin{array}{c} \sum_{j=1}^{r}\alpha_{j}^{\left(i\right)}=1,\alpha_{j}^{\left(i\right)}>0\\ {\tilde{X}}_{k-1}^{\left(i\right)}=\sum_{j=1}^{r}{\left(\mu_{k-1}^{j|i}\right)}^{2}X_{k-1}^{\left(j\right)}/\alpha_{j}^{\left(i\right)} \end{array} \end{array}$$

Based on the Lagrangian multiplier method, the optimal value of $\alpha_{j}^{\left(i\right)}$ is calculated by

(25) $$\alpha_{j}^{\left(i\right)}=\mu_{k-1}^{j|i}\sqrt{\mathrm{tr}(X_{k-1}^{\left(j\right)})}{\left(\sum_{j=1}^{r}\mu_{k-1}^{j|i}\sqrt{\mathrm{tr}(X_{k-1}^{\left(j\right)})}\right)}^{-1}.$$

### 4.2. Model-Conditioned Filtering

Then each subfilter for model $m_{k}^{i}$ updates state estimates with the new measurement $z_{k}$. From Equations (2) and (5), the prediction set of means can be given by

(26) $$\begin{array}{cc} X_{k,k-1}^{\left(i\right)} & =F_{k}^{\left(i\right)}ℰ({\tilde{x}}_{k-1}^{\left(i\right)},{\tilde{X}}_{k-1}^{\left(i\right)})⊕G_{k}^{\left(i\right)}D_{k}^{\left(i\right)}\\ & =ℰ(F_{k}^{\left(i\right)}{\tilde{x}}_{k-1}^{\left(i\right)},F_{k}^{\left(i\right)}{\tilde{X}}_{k-1}^{\left(i\right)}{\left(F_{k}^{\left(i\right)}\right)}^{T})⊕ℰ(0,G_{k}^{\left(i\right)}D_{k}^{\left(i\right)}{\left(G_{k}^{\left(i\right)}\right)}^{T}) \end{array}$$

The ellipsoid $ℰ({\hat{x}}_{k,k-1}^{\left(i\right)},X_{k,k-1}^{\left(i\right)})$ externally approximates the sum with

(27) $${\hat{x}}_{k,k-1}^{\left(i\right)}=F_{k}^{\left(i\right)}{\tilde{x}}_{k-1}^{\left(i\right)}$$

and

(28) $$X_{k,k-1}^{\left(i\right)}=(1+p^{-1})F_{k}^{\left(i\right)}{\tilde{X}}_{k-1}^{\left(i\right)}{\left(F_{k}^{\left(i\right)}\right)}^{T}+(1+p)G_{k}^{\left(i\right)}D_{k}^{\left(i\right)}{\left(G_{k}^{\left(i\right)}\right)}^{T}.$$

Equations (27) and (28) are obtained on the basis of **Lemma 2**. The parameter p that optimizes the trace of $X_{k,k-1}^{\left(i\right)}$ is

(29) $$p=\sqrt{\frac{\mathrm{tr}(F_{k}^{\left(i\right)}{\tilde{X}}_{k-1}^{\left(i\right)}{\left(F_{k}^{\left(i\right)}\right)}^{T})}{\mathrm{tr}(G_{k}^{\left(i\right)}D_{k}^{\left(i\right)}{\left(G_{k}^{\left(i\right)}\right)}^{T})}}.$$

The associated covariance matrix is stated by

(30) $$P_{k,k-1}^{\left(i\right)}=F_{k}^{\left(i\right)}{\tilde{P}}_{k-1}^{\left(i\right)}{\left(F_{k}^{\left(i\right)}\right)}^{T}+G_{k}^{\left(i\right)}Q_{k}^{\left(i\right)}{\left(G_{k}^{\left(i\right)}\right)}^{T}.$$

In the case of vanishing bounded perturbations, the state estimate is updated by means of the Kalman filter, as below:

(31) $$\begin{array}{cc} {\hat{x}}_{k}^{\left(i\right)} & ={\hat{x}}_{k,k-1}^{\left(i\right)}+K_{k}^{\left(i\right)}(z_{k}-H_{k}^{\left(i\right)}{\hat{x}}_{k,k-1}^{\left(i\right)})\\ & =(I-K_{k}^{\left(i\right)}H_{k}^{\left(i\right)}){\hat{x}}_{k,k-1}^{\left(i\right)}+K_{k}^{\left(i\right)}z_{k} \end{array}$$

(32) $$P_{k}^{\left(i\right)}=(I-K_{k}^{\left(i\right)}H_{k}^{\left(i\right)})P_{k,k-1}^{\left(i\right)},$$

where the gain $K_{k}^{\left(i\right)}$ is given by

(33) $$K_{k}^{\left(i\right)}=P_{k,k-1}^{\left(i\right)}{\left(H_{k}^{\left(i\right)}\right)}^{T}{\left(H_{k}^{\left(i\right)}P_{k,k-1}^{\left(i\right)}{\left(H_{k}^{\left(i\right)}\right)}^{T}+R_{k}^{\left(i\right)}\right)}^{-1}.$$

In the presence of set-membership uncertainty, the measurement $z_{k}$ combined with the ellipsoidal error can be regarded as an ellipsoid set,

(34) $$Z_{k}^{\left(i\right)}=\{z_{k}-e_{k}^{\left(i\right)}|e_{k}^{\left(i\right)}\in {ℰ}_{k}^{\left(i\right)}\}=ℰ(z_{k},E_{k}^{\left(i\right)}).$$

Then the predicted set $ℰ({\hat{x}}_{k,k-1}^{\left(i\right)},X_{k,k-1}^{\left(i\right)})$ is updated through

(35) $$\begin{array}{cc} X_{k}^{\left(i\right)} & =(I-K_{k}^{\left(i\right)}H_{k}^{\left(i\right)})ℰ({\hat{x}}_{k,k-1}^{\left(i\right)},X_{k,k-1}^{\left(i\right)})+K_{k}^{\left(i\right)}Z_{k}^{\left(i\right)}\\ & =(I-K_{k}^{\left(i\right)}H_{k}^{\left(i\right)})ℰ({\hat{x}}_{k,k-1}^{\left(i\right)},X_{k,k-1}^{\left(i\right)})+K_{k}^{\left(i\right)}ℰ(z_{k},E_{k}^{\left(i\right)})\\ & =ℰ((I-K_{k}^{\left(i\right)}H_{k}^{\left(i\right)}){\hat{x}}_{k,k-1}^{\left(i\right)},(I-K_{k}^{\left(i\right)}H_{k}^{\left(i\right)})X_{k,k-1}^{\left(i\right)}{\left(I-K_{k}^{\left(i\right)}H_{k}^{\left(i\right)}\right)}^{T})+ℰ(K_{k}^{\left(i\right)}z_{k},K_{k}^{\left(i\right)}E_{k}^{\left(i\right)}{\left(K_{k}^{\left(i\right)}\right)}^{T}) \end{array}$$

It is easily deduced that the shaping matrix of $ℰ({\hat{x}}_{k}^{\left(i\right)},X_{k}^{\left(i\right)})$, satisfying $ℰ({\hat{x}}_{k}^{\left(i\right)},X_{k}^{\left(i\right)})⊇X_{k}^{\left(i\right)}$, is

(36) $$X_{k}^{\left(i\right)}=(1+q^{-1})(I-K_{k}^{\left(i\right)}H_{k}^{\left(i\right)})X_{k,k-1}^{\left(i\right)}{\left(I-K_{k}^{\left(i\right)}H_{k}^{\left(i\right)}\right)}^{T}+(1+q)K_{k}^{\left(i\right)}E_{k}^{\left(i\right)}{\left(K_{k}^{\left(i\right)}\right)}^{T},$$

with the parameter q chosen to minimize the trace of $X_{k}^{\left(i\right)}$: This is

(37) $$q=\sqrt{\frac{\mathrm{tr}((I-K_{k}^{\left(i\right)}H_{k}^{\left(i\right)})X_{k,k-1}^{\left(i\right)}{\left(I-K_{k}^{\left(i\right)}H_{k}^{\left(i\right)}\right)}^{T})}{\mathrm{tr}(K_{k}^{\left(i\right)}E_{k}^{\left(i\right)}{\left(K_{k}^{\left(i\right)}\right)}^{T})}}.$$

### 4.3. Mode Probability Update

The mode likelihood is obtained as

(38) $$L_{k}^{\left(i\right)}≜p[{\tilde{z}}_{k}^{\left(i\right)}|m_{k}^{\left(i\right)},Z^{k-1}]\overset{assume}{=}N({\tilde{z}}_{k};0,S_{k}^{\left(i\right)}),$$

where ${\tilde{z}}_{k}=z_{k}-H_{k}^{\left(i\right)}{\hat{x}}_{k,k-1}^{\left(i\right)}$, $S_{k}^{\left(i\right)}=H_{k}^{\left(i\right)}P_{k,k-1}^{\left(i\right)}{\left(H_{k}^{\left(i\right)}\right)}^{T}+R_{k}^{\left(i\right)}$.

On this basis, we can calculate the model probability by

(39) $$\mu_{k}^{\left(i\right)}=\frac{\mu_{k|k-1}^{\left(i\right)}L_{k}^{\left(i\right)}}{\sum_{j}\mu_{k|k-1}^{\left(j\right)}L_{k}^{\left(j\right)}}.$$

### 4.4. Estimation Fusion

Using the matching model, the overall set of means can be expressed as

(40) $$X_{k}=\mu_{k}^{\left(1\right)}ℰ({\hat{x}}_{k}^{\left(i\right)},X_{k}^{\left(i\right)})⊕\mu_{k}^{\left(2\right)}ℰ({\hat{x}}_{k}^{\left(i\right)},X_{k}^{\left(i\right)})⊕\cdots ⊕\mu_{k}^{\left(r\right)}ℰ({\hat{x}}_{k}^{\left(i\right)},X_{k}^{\left(i\right)}).$$

Obviously the problem here is similar to that in Section 4.1, and thus the minimal-trace ellipsoid $ℰ({\hat{x}}_{k},X_{k})$ containing the sum $X_{k}$ is obtained based on Equations (20)–(25):

(41) $${\hat{x}}_{k}=\sum_{i}{\hat{x}}_{k}^{\left(i\right)}\mu_{k}^{\left(i\right)},$$

(42) $$X_{k}=\left(\sum_{j}^{r}\mu_{k}^{\left(i\right)}\sqrt{\mathrm{tr}(X_{k}^{\left(i\right)})}\right)\left(\sum_{j}^{r}\mu_{k}^{\left(i\right)}X_{k}^{\left(i\right)}/\sqrt{\mathrm{tr}(X_{k}^{\left(i\right)})}\right).$$

It should be noted that this solution is optimal only in the family of ellipsoids, as in Equation (23). The overall covariance is stated as

(43) $$P_{k}=\sum_{i}\left[P_{k}^{\left(i\right)}+({\hat{x}}_{k}-{\hat{x}}_{k}^{\left(i\right)})({\hat{x}}_{k}-{\hat{x}}_{k}^{\left(i\right)}{)}^{\prime }\right]\mu_{k}^{\left(i\right)}.$$

**Remark** **1.** *Finally, the proposed algorithm is implemented as below:*

***Step 1.** Initialize ${\hat{x}}_{0}$, $P_{0}$, $X_{0}$, and set k←1.*

***Step 2.** Model-conditioned reinitialization: Compute the mixing weight $\mu_{k-1}^{j|i}$ using Equations (18) and (19), and then for each sum filter, compute ${\tilde{x}}_{k-1}^{\left(i\right)}$, ${\tilde{P}}_{k-1}^{\left(i\right)}$, and ${\tilde{X}}_{k-1}^{\left(i\right)}$ using Equations (20), (21), and (23), respectively.*

***Step 3.** Model-conditioned filtering: For each subfilter, update ${\hat{x}}_{k}^{\left(i\right)}$, $P_{k-1}^{\left(i\right)}$, and $X_{k-1}^{\left(i\right)}$ using Equations (27)–(33) and (36).*

***Step 4.** Mode probability update: Calculate the model probability using Equation (39).*

***Step 5.** Estimation fusion: Using the matching model, calculate the overall estimate results ${\hat{x}}_{k}$, $X_{k}$, and $P_{k}$ using Equations (41)–(43), respectively.*

***Step 6.** Set k←k+1 and return to Step 2.*

## 5. Experiments and Results

### 5.1. Experimental Setup

**Figure 4:**
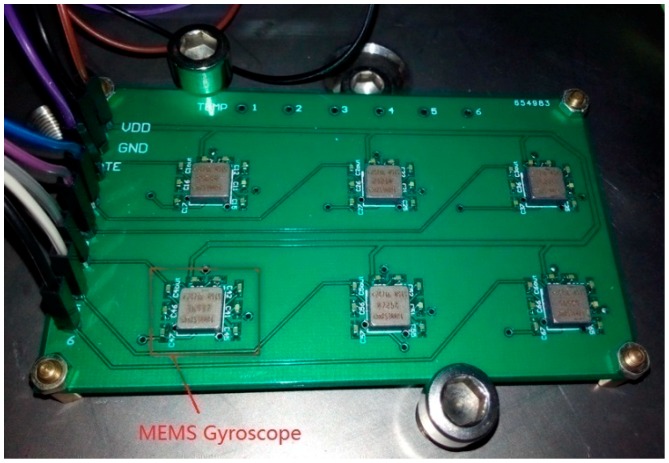
Prototype of the gyro array.

In this section, experiments were implemented to demonstrate the performance of the proposed filter. Six MEMS gyroscope chips (ADXRS300 [27]) with external circuits were soldered onto the same printed circuit board (PCB) to construct an array. The prototype of the gyro array is shown in Figure 4. The output signals of the gyroscopes were collected through the data acquisition module constructed by a PCI (peripheral component interconnection) extension for instrumentation (PXI) system. The bandwidth of an individual gyroscope was 40 Hz, so the sampling rate was set to be 200 Hz to satisfy the Nyquist theorem.

In the experiment, the gyro array was put on a horizontal turntable. The experiment lasted for 180 s, during which the turntable was set to rotate as follows:

- 15–40 s: Swing with 10° angle amplitude and 0.5 Hz frequency;
- 45–65 s: Rotate with a 40°/s constant rate;
- 70–90 s: Rotate with a −20°/s constant rate;
- 100–136 s: Swing with 10° angle amplitude and 0.25 Hz frequency;
- 145–172 s: Swing with 20° angle amplitude and 0.5 Hz frequency.

**Figure 5:**
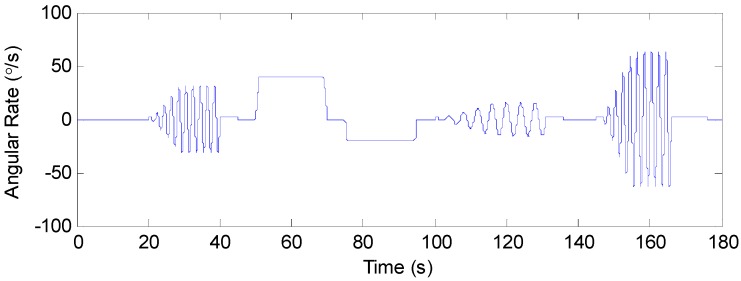
The true angular rate.

During the other times, except for the above time periods, the turntable kept still. It should be noted that it took a period of time to adjust the motion state each time for this turntable. Thus, both the start and end phases of each motion state had transition periods, and only the middle part was in accordance with the set conditions above. The true angular rate of the turntable is shown in Figure 5. 

Then the rate signals of the gyroscopes in the array were gathered and processed. 

Through multiple tests, the correlation factor between the component gyroscopes was obtained and is shown in Table 1: It played an important role in accuracy improvement of the gyro array [28]. This meant the elements in CorrM were given. The correlation factor was analyzed with static rate signals for 1 h, and the computing method of the correlation factor followed [14].

Four models were used in the experiment, and other parameters in the model were given as ΔT=0.005, r=0.6, and e=0.6. $Q_{i}$ and $D_{i}$ for different models are shown in Table 2.

### 5.2. Results

**Figure 6:**
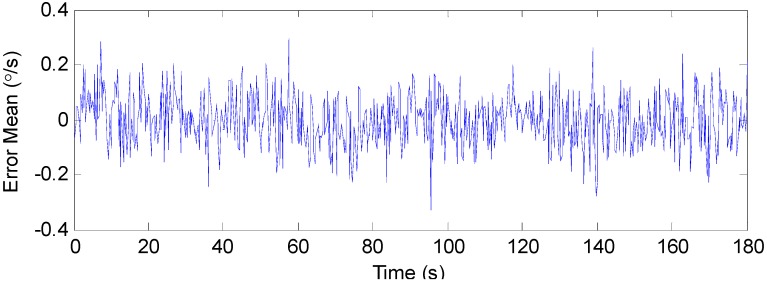
The error mean over different time periods.

For comparison purposes, the stand IMM estimator using a Kalman filter and the combined filter in Section 4.2 using a single model were implemented. First, the output errors of one of the individual gyroscopes were analyzed. By using a sliding window with a width of 50, the means of the error over different time periods were calculated, as shown in Figure 6. It can be seen that the error mean value of the gyroscope changed significantly with time due to the dynamic change of input. This indicated that the assumption of two random variables as noise representation in this paper was reasonable, because a set of means was the main feature distinguishing this assumption from the single stochastic uncertainty assumption. In addition, it can be seen that the means of different time periods were all within the hypothetical set constructed by the parameters given in Section 5.1. 

**Figure 7:**
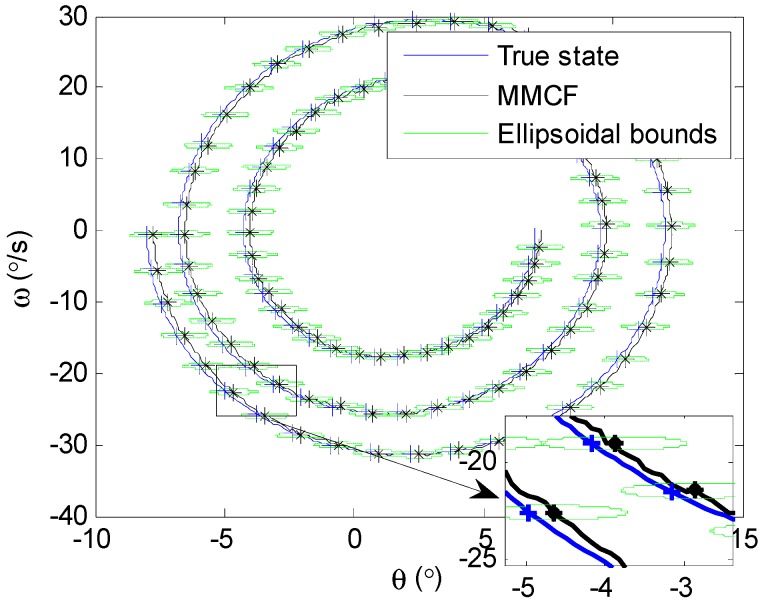
Phase-plane estimation using the MMCF.

**Figure 8:**
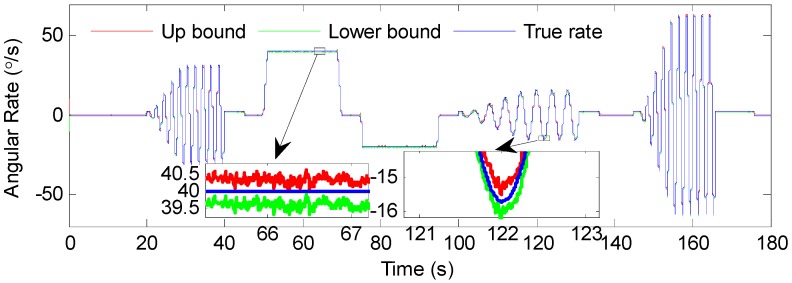
The estimated bounds by MMCF.

Based on this assumption, the proposed multi-model combined filter could get an ellipsoidal feasible set of means at each moment (i.e., $ℰ({\hat{x}}_{k},X_{k})$), while the standard IMM filter with respect to Gaussian noise could only obtain a point estimate. In addition, the center of the ellipsoid ${\hat{x}}_{k}$ could be considered to be the point estimate when needed. Figure 7 shows the state ellipsoidal estimates from 25 s to 30 s in the phase plane using the proposed algorithm. It should be noted that, for clarity, only part of the ellipsoids were selected to be shown in the figure, and the time interval between two adjacent ellipsoids was 0.05 s. The center of each ellipsoid is highlighted with “*”, and the true state at the corresponding moment is highlighted with “+”. It can be seen from Figure 7 that all of the values of the true state at every moment lay within the corresponding ellipsoid. On this basis, the upper and lower boundaries of the estimation were acquired by using the shape matrices of ellipsoids, as shown in Figure 8. It can be guaranteed that the angular rate estimates are within a hard boundary and that the bounded sets contain the true value, which is meaningful for the robustness of the system in control and guidance applications. 

**Figure 9:**
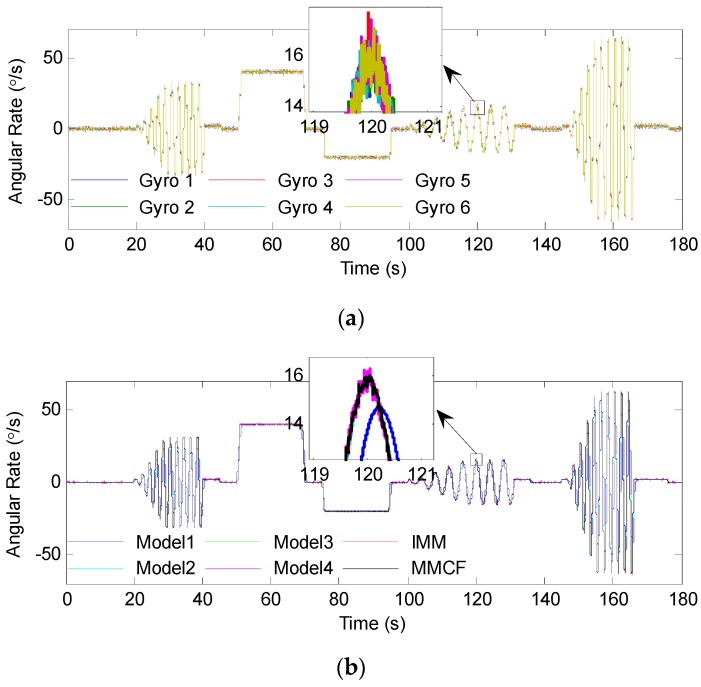
The overall experiment outputs: (**a**) The outputs of individual gyroscopes; (**b**) the angular rate of the gyro array estimated by using different methods.

**Figure 10:**
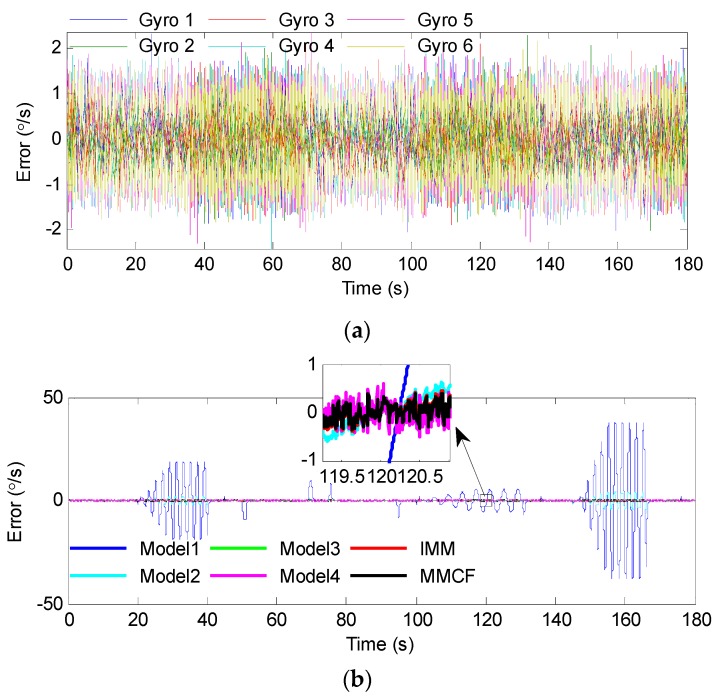
Errors of the overall outputs: (**a**) The output errors of individual gyroscopes; (**b**) the angular rate errors of the gyro array estimated by using different methods.

**Figure 11:**
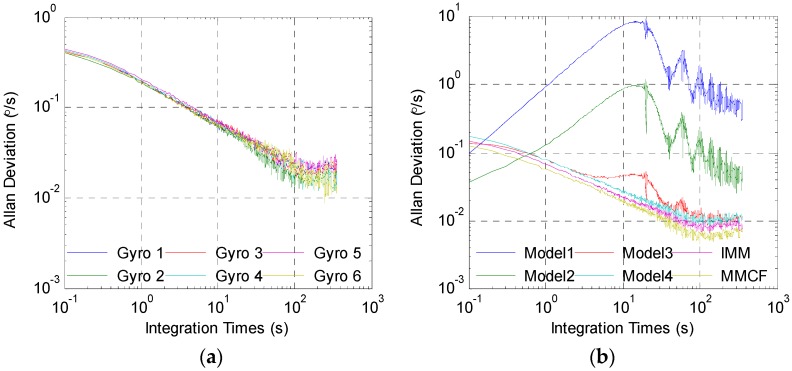
The Allan deviation test results: (**a**) The Allan deviation of the errors from individual gyroscopes; (**b**) the Allan deviation of the errors from the gyro array.

Below, the center of the ellipsoid was used as the point estimate, and the accuracy of the gyro array was analyzed. The original rate signals of the individual gyroscopes and processing results by different methods are given in Figure 9. The output errors and estimated errors are shown in Figure 10. In order to demonstrate the effect of the above filters clearly, the root mean square error (RMSE) of the estimated results was calculated and is presented in Table 3. For further tests, the errors were analyzed through the Allan deviation method, and the results are shown in Figure 11. Besides, the angular random walk (ARW), rate random walk (RRW), and bias instability (BI) were calculated from an Allan deviation plot, and are also presented in Table 3. In the Allan deviation test, the measurements were recorded for 1 h at a sampling rate of 200 Hz. Before that, the gyroscopes were powered on for 0.5 h. The experiments were performed in a temperature control turntable, and the temperature was set to be 25 °C. Other experimental configurations, such as hardware setup, were the same as described in Section 5.1. The turntable kept still during most times, except for 1 min. The turntable was set to swing with 10° angle amplitude and 0.5 Hz frequency in this minute to test the dynamic performance of the proposed method. Then the difference between the output of the gyroscopes (array) and the actual rotational speed of the turntable was analyzed as the output error.

In Table 3, IF is the improve factor, defined as

(44) $$IF={\mathrm{RMSE}}_{s}/{\mathrm{RMSE}}_{a},$$

where ${\mathrm{RMSE}}_{s}$ refers to the RMSE of the rate signal for the single gyroscopes in the array, and ${\mathrm{RMSE}}_{a}$ is that of the gyro array.

It can be seen from these results that both the multi-model methods and the combined filter using some single models (Models 3 and 4) were effective in combining numerous gyroscopes, and the multi-model methods performed better. When using Model 1 or Model 2, the errors could be well suppressed by the filter when the input signal was 0 or constant. However, when the turntable swung, the filter led to amplitude attenuation, which demonstrated that this model could not accurately reproduce the dynamic characteristics of the input signal. When using Model 3 or Model 4, the result was the opposite: The errors were greatly reduced with sinusoidal rate input, but the filter had poor performance with constant rate input. Through switching the models according to different situations, the multi-model methods could reflect the dynamic characteristic of the input signal more accurately. Table 3 shows that the RMSE was reduced from 0.4916°/s to 0.1628°/s and 0.1478°/s by the IMM filter and MMCF, respectively. It reveals that the performance of the MMCF was higher than that of the IMM filter. From the Allan deviation plot and the results in Table 3, the ARW noise, RRW noise, and bias instability were all reduced by using the multi-model methods and combined filter with Models 3 and 4 to fuse measurements from the gyro array. In addition, it is obvious that the reduction factor by the MMCF was greater than that by other methods. As for the filter using Models 1 and 2, the above three kinds of noise were difficult to calculate due to large sinusoidal errors. The reason for the failure of Models 1–3 in Figure 11 was that these models could not accurately reproduce the dynamic characteristics of the input signal, and the filters using these models led to amplitude attenuation when the turntable swung. This means large sinusoidal errors were added to the output noise, similarly to the errors in Figure 10.

**Figure 12:**
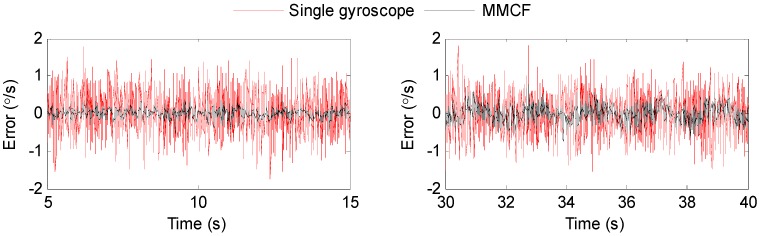

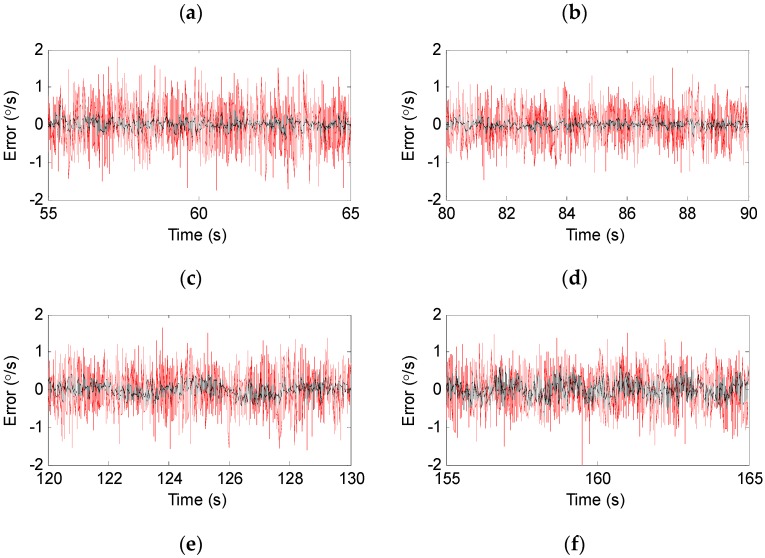
Estimated error with MMCF over different time periods: (**a**) at 5–15 s, the input signal was ω=0; (**b**) at 30–40 s, the input signal was ω=10πsin(πt)°/s; (**c**) at 55–65 s, the input signal was ω=40°/s; (**d**) at 80–90 s, the input signal was ω=−20°/s; (**e**) at 120–130 s, the input signal was ω=5πsin(0.5πt)°/s; and (**f**) at 155–165 s, the input signal was ω=20πsin(πt)°/s.

To further analyze the performance of the proposed method, the estimated errors over different time periods are shown in Figure 12. The detailed results are illustrated in Table 4. 

Figure 12 shows that the proposed method could effectively reduce the errors of the gyroscope in any kind of motion. The statistics results in Table 4 also demonstrate the performance improvements. It can be seen from Table 4 that the RMSEs of the estimation were obviously reduced compared to the original rate signals of the individual gyroscopes. Furthermore, the performance of the proposed method had to do with the dynamic characteristics of the input signal. The reduction factor with respect to 0 input and constant input is about 4–5, whereas the corresponding factor for sinusoidal input was about 2–3, which was lower than that of the 0 input and constant input. Additionally, it indicated an increase in the RMSE with increasing frequency and amplitude with respect to the swing rate signal.

## 6. Conclusions

In this paper, a multi-model combined filter allowing for simultaneous treatment of stochastic and set-membership uncertainties was proposed to combine multiple MEMS gyroscopes to improve overall accuracy. The dual uncertainties model of a gyroscope array was established. To reproduce the dynamic characteristics of the rate signal, multiple models with different process noises were involved in the filter. Combined with the IMM update process and the calculation method for a Minkowski sum of multiple ellipsoidal sets, the multi-model state estimates integrating dual uncertainties was updated. The experimental results showed that the RMSE of the estimated rate signal by the proposed method was reduced from 0.4916°/s to 0.1478°/s, which performed better than the IMM filter and combined filters with single models. This proved that the proposed method is efficient in improving the system overall performance and that it produces better adaptability to different kinds of uncertainties and different dynamic characteristics. Moreover, the proposed method can provide the bounds of the rate signal estimates. This property is meaningful for the robustness of the system and benefits attitude control and guidance.

## Figures and Tables

**Table 1:** Correlation factor between the component gyroscopes.

	Gyro 1	Gyro 2	Gyro 3	Gyro 4	Gyro 5	Gyro 6
**Gyro 1**	1.0000	0.0016	−0.0047	0.0297	0.0467	0.0451
**Gyro 2**	0.0016	1.0000	0.0595	−0.0095	0.0591	0.0542
**Gyro 3**	−0.0047	0.0595	1.0000	−0.0939	−0.0381	−0.0688
**Gyro 4**	0.0297	−0.0095	−0.0939	1.0000	0.0169	0.0946
**Gyro 5**	0.0467	0.0591	−0.0381	0.0169	1.0000	0.0501
**Gyro 6**	0.0451	0.0542	−0.0688	0.0946	0.0501	1.0000

**Table 2:** $Q_{i}$ and $D_{i}$ for different models.

Models	Model 1	Model 2	Model 3	Model 4
Parameters	$Q_{1}=8$ $D_{1}=6$	$Q_{2}=80$ $D_{2}=60$	$Q_{3}=800$ $D_{3}=600$	$Q_{4}=8000$ $D_{4}=6000$

**Table 3:** The overall results of the gyro array. IMM: Interacting multiple model; RMSE: Root mean square error; IF: Improve factor; ARW: Angular random walk; RRW: Rate random walk; BI: Bias instability.

Terms	Single Gyroscope	Model 1	Model 2	Model 3	Model 4	IMM	MMCF
**RMSE** (°/s)	0.4916	8.3886	0.9663	0.1828	0.2009	0.1628	0.1478
**IF**	1	0.0586	0.5087	2.6893	2.4470	3.0197	3.3261
**ARW** (${}^{\circ}/h^{1/2}$)	11.4362	/	/	4.8251	4.8458	4.0748	3.4395
**RRW** (${}^{\circ}/h^{3/2}$)	435.2964	/	/	192.5314	198.8246	178.2495	156.2842
**BI** (°/h)	63.2521	/	/	41.5448	33.1668	28.9512	21.0312

**Table 4:** The test results over different time periods (different input).

Time (s)	Input (°/s)	Single Gyroscope	Gyro Array	
		RMSE (°/s)	RMSE (°/s)	*IF*
5–15	0	0.4939	0.1120	4.4098
30–40	ω=10πsin(πt)	0.4775	0.2081	2.2946
55–65	40	0.5190	0.1212	4.2822
80–90	−20	0.4320	0.0850	5.0824
120–130	ω=5πsin(0.5πt)	0.4778	0.1686	2.8339
155–165	ω=20πsin(πt)	0.4797	0.2200	2.1805

Note: *t* refers to the time.
